# Utilization of qPCR and ELISA Tests to Detect *Cytauxzoon felis* (*Theileriidae*) in Domestic Cats (*Felis catus*) from South Central USA

**DOI:** 10.3390/vetsci13050426

**Published:** 2026-04-28

**Authors:** Ryan Carson, Sarah Myers, Catlyn Ballard, Ruth Scimeca

**Affiliations:** Department of Veterinary Pathobiology, College of Veterinary Medicine, Oklahoma State University, Stillwater, OK 74074, USA

**Keywords:** *Cytauxzoon felis*, ELISA, diagnostic tests

## Abstract

*Cytauxzoon felis* is a tick-borne protozoan parasite of considerable importance to domestic cats in the United States. Despite high mortality among clinically presenting cats, some cats may either recover from infection, becoming permanent chronic carriers of *C. felis*, or may not develop disease following exposure. *C. felis* in healthy (asymptomatic) cats is currently detected by qPCR or through observation of piroplasms in blood smears. The present study aims to compare the prevalence of *C. felis* in healthy populations of pet and free-ranging cats by using two different diagnostic tests: an indirect ELISA and a probe-based qPCR. Samples, along with demographic information, were collected collaboratively from healthy cats in the states of Oklahoma, Arkansas, and Missouri (USA) in either pet or free-range populations. Significantly more cats were detected to be *C. felis* positive by the ELISA than qPCR. Of the total of cats tested by ELISA and qPCR, 248 (35.37%) tested ELISA positive but qPCR negative, indicating formation of specific *C. felis* antibodies in cats with undetectable circulating *C. felis* DNA. The results of this study provide insights into the occurrence of *C. felis* in healthy cats that are exposed in the south-central United States and present a useful diagnostic strategy for detecting *C. felis*.

## Simple Summary

Bobcat fever, or Cytauxzoonosis, is a tick-transmitted parasitic disease that infects felids. Domestic cats become severely ill when bitten by infected lone star ticks carrying the parasite. *Cytauxzoon felis* is the causative agent, which circulates in the blood of felids. Bobcats (*Lynx rufus*) and domestic cats that survive the acute phase of infection act as reservoirs. Acutely infected cats become less active, have reduced food and water intake, and experience pain and fever. If not treated promptly, cats succumb to the disease. The diagnostic method used to detect this parasite is qPCR, which is a very sensitive method that identifies cats with circulating parasites in the blood by detecting the parasite’s DNA. This study aimed to utilize a combination of diagnostic methods in a healthy population of cats to evaluate the prevalence of possible exposure and infection in an area where bobcat fever is commonly diagnosed.

## 1. Introduction

*Cytauxzoon felis* is a protozoan parasite responsible for causing Cytauxzoonosis, or bobcat fever. It belongs to the family Theileiriidae and is transmitted to felids by *Amblyomma americanum*, the lone star tick. The life cycle of *C. felis* initiates when an infected tick feeds on a naïve cat; the tick then transmits the sporozoite infective stage to the cat. Within the vertebrate host, *C. felis* initially replicates in the mononuclear cells and undergoes schizogony, which leads to schizont formation in the infected cell [1,2]. This initial replicative stage occurs in the endothelium or lumina of veins and venules and in the interstitial space of the spleen and lymph nodes, corresponding to the acute stage of the infection, where schizonts can block and occlude blood vessels. Schizonts end up erupting and releasing merozoites, which invade erythrocytes, becoming piroplasms [3]. This last stage remains throughout the life of the recovered infected felid and corresponds to the source of infection for lone star ticks. Domestic cats can become severely ill, with clinical signs including lethargy, anorexia, fever, dehydration, elevation of the third eyelid, vocalization due to abdominal pain, and icterus. If cats are not promptly treated, they can become hypothermic and succumb to the infection; cats who recover remain chronically infected with no clinical signs observed during this stage. In addition to domestic cats, other felids, including bobcats, can become infected and are considered a reservoir for the parasite in the United States [4]. *C. felis* is the only known piroplasmid that infects domestic cats in the USA, and although its prevalence is almost limited to the lone star distribution area, it has high mortality rates among domestic cats. Several prevalence studies in bobcats have been published throughout the years, but little is known about morbidity and mortality in these reservoir hosts. In the past decade, more case reports of recovered infected cats have been published, and a higher number of cats are chronically infected but with no clinical signs of *C. felis* (hereinafter referred to as healthy cats). Detection methods employed to date are detection of the parasite by blood smear examination, conventional PCR, qPCR, and ddPCR [1,3,5]. An innovative ELISA test to detect IgM and IgG antibodies in acutely and chronically infected cats, by targeting a *C. felis*-specific immunogenic transmembrane protein (c88), was developed in 2022 [6]. Although a limited number of cats were enrolled in that study, it provided a different approach to evaluate the prevalence of *C. felis* in cats and indicated that IgM antibodies are detected earlier in the infection process than IgG. Therefore, by using the IgG ELISA test in addition to the observation of piroplasms in blood and molecular methods, we can now evaluate the occurrence of *C. felis* in healthy cats. This study aimed to compare the prevalence of *C. felis* in healthy cat populations of (i) pet cats and (ii) free-range cats from endemic areas of the country, including the states of Oklahoma, Arkansas, and Missouri, by using two different diagnostic tests: an indirect ELISA (IgG) and a probe-based qPCR.

## 2. Materials and Methods

### 2.1. Animal Samples

#### 2.1.1. Free-Range Cat Samples

Whole-blood and serum samples previously collected from 608 free-range cats were presented to Operation Catnip—the trap, neuter, release program at Oklahoma State University. Samples were collected in 2014 and 2016 and stored at −80 °C until processing. The demographic information obtained from this group was gender and age categorized as <6 months, 6 to 12 months, 1 to 3 years, or >3 years.

#### 2.1.2. Pet Cat Samples

Whole-blood and serum samples obtained from client-owned cats (*N* = 100) were collected by veterinary personnel from veterinary clinics located in the states of Arkansas, Oklahoma, and Missouri (USA) from April 2024 to August 2024. Pet owners filled out a signed consent form that included gender and age, using the same categories as for the free-range cats described above: lifestyle, described as indoor only, indoor/outdoor, or outdoor only; environment, classified as rural (population < 25,000), suburban (population 25,000 to 50,000), or urban (population > 50,000); and history of tick prevention use (Supplementary Figure S1). The inclusion criteria specified that cats should not show clinical evidence of disease.

#### 2.1.3. Experimentally Infected Cats

Five pathogen-free cats housed at Oklahoma State University (OSU) Animal Resources (AR) were utilized for blood and serum collection at day 0, before tick infestation and *C. felis* infection, and at 8, 12, and >42 days post-tick infestation (post *C. felis* infection) as previously described by Reichard et al. [7]. Samples were opportunistically used as part of another study (unpublished data) to evaluate antibody levels over time and considered late infection when collected after day 42 post-tick infestation. The samples from this group of cats were also tested by qPCR and blood smear examination.

#### 2.1.4. Control Samples

Whole-blood and serum samples from two cats experimentally infected with *C. felis* available from a previous study [5,7] were utilized as positive controls for the IgG ELISA, and two pathogen-free cats housed at OSU-AR from another study were utilized as negative controls.

### 2.2. Cytauxzoon felis Detection

#### 2.2.1. Indirect ELISA

The recombinant transmembrane protein contig 00088:95434-96586(-) (c88 rProtein) previously recognized as having high seroreactivity against *C. felis* was synthesized by GenScript Biotech (Piscataway, NJ, USA) [6,8] and cloned into the pET30 vector with a His tag for protein expression in *E. coli.* The cells were centrifuged and evaluated by Western blotting and SDS-PAGE to verify the molecular weight of the protein. The indirect ELISA previously described by Kao et al., 2022, was utilized to detect *C. felis*-specific IgG antibodies in serum samples from domestic cats [6]. A total amount of 100 μL of c88 rProtein in 0.1 < carbonate buffer (IgG: 2.5 μg/mL) was used to coat each well of 94-well flat-bottom polystyrene high bind micro-plates (Corning Inc. Glendale, AZ, USA) and incubated at 4 °C for 16 h. Next, the coating solution was removed, and the plate was blocked for 4 h using 2% bovine serum albumin in TEN buffer (0.1 M Tris-Cl, 0.01 M EDTA, 1 M NaCl, 200 μL/well). The blocking solution was then discarded, and the samples were diluted to a ratio of 1:500 in ELISA diluent (4% *v*/*v* fetal bovine serum, 0.5% *v*/*v* Triton X-100, and 2% bovine serum albumin in TEN buffer). A total amount of 100 μL of sample per well was added, with each plate including a positive, negative, and assay buffer control. The plate was incubated at 37 °C for one hour and further washed with 200 μL/well 0.2% Tween in TEN buffer five times.

Horseradish peroxidase (HRP)-conjugated goat anti-feline IgG-Fc fragment (Bethyl Laboratories, Inc., Montgomery, TX, USA, in-house conjugation performed with lynx rapid HTP antibody conjugation kit, Bio-Rad Laboratories, Hercules, CA, USA) was diluted to a ratio of 1:40,000 in ELISA diluent and added into wells (100 μL/well). After incubation at 37 °C for one hour, the plate was washed 5 times with 0.2% Tween in TEN buffer. Next, 100 μL of 1-Step™ Ultra TMB-ELISA substrate solution (ThermoFisher™Scientific. Waltham, MA, USA) was added per well and incubated at room temperature for 10 min. Lastly, 75 μL/ELISA stop solution (ThermoFisher™Scientific. Waltham, MA, USA) was added to complete the reaction. Absorbance was then measured at 450 nm using a SpectraMax^®^ Plus 384 Microplate Reader (Molecular Devices, LLC. San Jose, CA, USA). All samples were tested in duplicate.

#### 2.2.2. DNA Isolation and Probe-Based qPCR

Whole-blood samples collected in EDTA tubes were utilized for DNA isolation. The ReliaPrep™ Blood gDNA Miniprep System (Promega, Madison, WI, USA) was used following the manufacturer’s instructions. The extracted DNA was stored at −20 °C until further use. A total of 10 to 50 ng of DNA was utilized to amplify a 190-base-pair fragment of the cytochrome *c* oxidase subunit III (*cox3*) gene using qPCR as previously described [5]. Each reaction contained 10 µL of IDT PrimeTime^®^ Gene Expression Master Mix (Coralville, IA, USA); 0.5 µL of primers COX3F: 5′ CTA CAC TCT TTA CAC GTT TGT G 3′, COX3R: 5′ CGA AAT GCC AGT ATA CTC CT 3′, and probe 5′ HEX/TGA GTT TGC/ZEN/AAG GGC CAT TAT AAC ACC/31AbkFQ 3′; and 2 µL of sample and molecular-grade water up to 20 µL. All samples were run in duplicate using the CFX96 real-time thermocycler (Bio-Rad, Hercules, CA, USA). Thermocycling conditions included a polymerase activation step at 95 °C for 3 min, followed by 45 cycles of a 15 s denaturation step at 95 °C and a 30 s annealing–extension step at 60 °C.

### 2.3. Statistical Analysis

Absorbance data from an indirect IgG ELISA was plotted on Microsoft Excel 16.16.27 software (Redmond, WA, USA) to calculate the mean, standard deviation, and percentage of coefficient of variation (% CV) between duplicate samples within the same plate to ensure acceptable intra-plate consistency (% CV < 10%). Each sample was run in duplicate to obtain the mean absorbance values. The mean absorbance value for each sample was transformed into percent positivity (PP) using a previously published formula [9,10,11] to reduce inter-plate variability. The cut-off value was calculated by multiplying the standard deviation above the mean OD of the negative control samples three times. Statistical analysis was performed using GraphPad Prism V.10.6.1 (Dotmatics). Non-parametric tests were analyzed between groups using the Chi Square or Fisher’s tests, as applicable, to determine the significance of differences in the ELISA or qPCR prevalences across different sexes, ages, environments, lifestyles, or tick prevention uses, with *p* < 0.05 indicating a statistically significant difference.

## 3. Results

### 3.1. Free-Range Cats

A total of 601 free-range cats were tested by ELISA and qPCR. An additional group of seven cats did not have whole blood available and therefore only underwent an ELISA test. In this group of cats, 262 (43.1%) were determined to be *C. felis* positive by the ELISA, while qPCR detected 25 (4.2%) positive cats. Only 11 (1.8%) samples tested positive by the two methods, while 247 (41.1%) were positive by ELISA only, and 14 (2.3%) were qPCR positive but not detected by ELISA (Table 1).

### 3.2. Pet Cats

From the 100 samples tested in this group of cats, 4 (4%) were ELISA positive and 12 (12%) were detected by qPCR (Table 2). The two methods detected *C. felis* in three of these cats (3%), qPCR only detected it in nine (9%), and ELISA test only detected it in one (1%). No significant differences were observed among cats according to state.

#### 3.2.1. Lifestyle

The results demonstrated that the lifestyle of the cat was significant in the detection of *C. felis* by IgG ELISA (*p* = 0.046), but not when using the qPCR test. The prevalence of cats with outdoor access was 4% for the ELISA test, while no cats with an indoor lifestyle were detected as positive (Table 2).

#### 3.2.2. Environment

No significant results were detected in the ELISA test; however, the qPCR was statistically significant (*p* = 0.016), with the highest percentage of detection (3%) observed in cats from rural areas; no cats living in suburban areas were detected as positive, and 1% of cats living in urban areas were qPCR positive.

#### 3.2.3. Tick Prevention

The use of tick prevention did not reveal any significant result, yet percentages of detection by qPCR were higher in cats with no history of prevention. Interestingly, ELISA prevalence was higher in cats with tick prevention (Table 2).

### 3.3. Free-Range and Pet Cats

Significant statistical differences between free-range cats and pets were detected by both ELISA (*p* < 0.0001) and qPCR (*p* = 0.0012) tests, with more positive cats detected in the free-range population (Supplementary Figure S2).

#### 3.3.1. Gender

No significant differences were obtained when comparing gender and *C. felis* detection by either method.

#### 3.3.2. Age

Significant results were found when analyzing the ELISA results and age of cats, with a higher percentage of detection (17.7%) observed in cats aged 1 to 3 years old (*p* = 0.0396). No significant results were obtained between qPCR detection and age of cats.

### 3.4. Experimentally Infected Cats

Four cats were detected positive by qPCR, by observation of piroplasms in the blood smear and by clinical signs consistent with *C. felis* infection after tick feeding. In three of these qPCR-positive cats, the ELISA PP values increased on average from 20.2 at day 12 PI to 86.4 at day 42 PI (Supplementary Table S1; Supplementary Figure S3).

## 4. Discussion

Although geographically limited by the distribution of its vector, the lone star tick, cytauxzoonosis is considered highly pathogenic with elevated mortality rates in domestic cats [2]. Because cats infected with *C. felis* are usually detected in veterinary clinics when cats are symptomatic and in advanced stages of the disease, the assumption is that most cats exposed to ticks infected with *C. felis* will develop clinical signs [1,12,13]. However, besides the qPCR test used to detect the presence of *C. felis* DNA, no tests have been used to detect the prevalence of cats that might have been exposed but are not actively infected. In this study, we utilized an ELISA-based method [6] to evaluate the prevalence of cats with *C. felis*-specific IgG antibodies and compared the same cat samples by using a probe-based qPCR. As initially suspected, a lower percentage of cats were detected as positive by qPCR when compared to the ELISA method (Table 1 and Table 2). The prevalence rate detected by qPCR in the cats tested in this study was similar to previous studies [14,15], with the majority of samples detected as positive by qPCR in pet cats from Arkansas (*N* = 10), where higher prevalence rates have been reported [16]. Only two pet cats from Oklahoma were qPCR positive. Yet, a new insight was obtained by utilizing the indirect ELISA test; we learned that a high percentage of cats have detectable IgG antibody levels (Table 1), which could indicate that (i) cats are being exposed to ticks infected with the protozoan parasite but not becoming actively infected, or (ii) cats become actively infected, recover from the infection, and have undetectable parasitemia by qPCR. We believe that this last scenario is less likely to occur based on experimental infections where cats have become infected without developing severe clinical signs but remained parasitemic, as seen in one of the experimentally infected cats included in this study (cat 3, Supplementary Table S1), which became infected without developing clinical signs. This cat was detected as positive at day 40 PI by qPCR and by observation of piroplasms in the blood smear examination; IgG antibodies for *C. felis* were detected at day 42 PI.

Cats could experience antibody formation without developing clinical signs due to low parasitemia in the infected ticks, which might be the case in experimentally infected cat 3, or in natural infections due to short feeding times of the infected tick and removal by grooming. Further studies to evaluate exposure to *C. felis*-infected ticks over short periods of time could be helpful in assessing if IgG antibody formation is detectable by the ELISA test, without developing clinical signs and parasitic replication. When we compared free-range cats and pet cats, statistically significant results were obtained, indicating that most of the ELISA-positive cats were from the free-range population. We must highlight that samples from the two cat populations were taken during different periods of time and therefore this comparison should be considered tentatively. Furthermore, within the pet population, the prevalence of ELISA was higher in cats with an indoor/outdoor lifestyle (Table 2). In 2020, a study from eastern Kansas reported a *C. felis* prevalence of 25.8% in asymptomatic pet cats, which is the highest prevalence reported in the United States. The authors of this study utilized qPCR to detect the infection, compared to previous studies where conventional or nested PCR was utilized [17]. Because qPCR generally has a higher sensitivity level than conventional PCR, this could explain the higher percentage of detection reported.

When assessing the cats that tested positive using both methods, the prevalence was only 1.8% for the free-ranging cats and 3% for the pet cats. This result could be explained by the fact that pet cats usually receive veterinary treatment more often and promptly than free-range cats, or by the differences in tick activity and *C. felis* infection rates during those two periods of time. We assumed that cats detected as positive by both methods recovered from the infection and maintained detectable antibodies. However, we also detected a group of cats that tested positive by qPCR, but no antibodies were detected by the ELISA (Table 1 and Table 2); this result could be explained by low antibody production in some cats, which was also detected in one of the samples from the experimentally infected cats included in this study (cat 1, Supplementary Table S1). Although it was determined positive by qPCR at day 8 PI and displayed clinical signs, this cat did not develop detectable antibodies by day 42 PI. Cat 1 was adopted after recovery at day 45 PI and removed from the study immediately after; no follow-up was performed to evaluate whether antibodies would be detected in later stages of the chronic infection (Supplementary Table S1). This combined information from naturally and experimentally infected cats might indicate that some cats with lower antibody levels are susceptible to developing a newly acquired *C. felis* infection, even though they remain parasitemic from the first infection, as previously reported in a naturally infected cat [18], but this has not been proven experimentally. A recent report from cats naturally infected in the state of Indiana included a cat identified as a survivor with no history of clinical cytauxzoonosis [19], indicating that some naturally infected cats can develop subclinical infections and remain parasitemic [20].

From the pet population analysis, we were also able to collect information regarding the use of tick prevention; while no statistically significant results were found, eight cats were positive by qPCR with no history of prevention. These cats pose a risk by acting as reservoirs, and these findings highlight the importance of testing cats in endemic areas to avoid local outbreaks [12]. The use of topical acaricidal formulations has previously been shown to be effective at preventing the transmission of *C. felis* to cats infested with *Amblyomma americanum*, reinforcing the importance of routine acaracide use for any pet cats at risk, especially in areas with unprotected cat populations serving as a reservoir [21].

The prevalence of detection using either method and the correlation with age and sex did not reveal significant findings in the latter, but ELISA detected a significantly higher percentage (17.7%) of cats positive between the ages of 1 and 3 years old, possibly related to the activity of the cats, tick exposure, and a generally good immune response at that age range compared to younger or older cats [22]. Seasonality was not analyzed because samples from free-range cats were collected between January and April and between September and November, while pet samples were collected during the summer months. An additional limitation of this study is that samples from free-range cats were collected from 2014 to 2016 and opportunistically used, while the samples from the pet cats were collected in 2024. This gap in time hinders an accurate conclusion on the current *C. felis* prevalence in free-range cats in the state of Oklahoma; therefore, further studies should be conducted with samples collected throughout the year to evaluate seasonality and current prevalence. Because the time of collection and the number of pet cat samples analyzed in this study were limited, the analysis performed here was intended to provide preliminary information that can be further tested to provide specific conclusions. Additionally, testing experimentally infected cats or pets over time could provide information on antibody levels after infection or tick exposure.

## 5. Conclusions

While qPCR is useful in detecting chronic carrier cats, it is limited to detecting the DNA of circulating parasites. The results of this study, which used the ELISA method in addition to qPCR, suggest that some cats could be exposed to ticks infected with *C. felis* but do not develop an active infection. Therefore, utilizing a combination of diagnostic tests helps in identifying endemic areas or even households where cats are likely to be exposed to *C. felis*-infected ticks and where tick prevention should be emphasized. Further studies evaluating antibody levels over time in experimentally infected cats or naturally infected survivor cats should be conducted, in combination with qPCR.

## Figures and Tables

**Table 1:** Total of free-range cats tested for *Cytauxzoon felis* infection by indirect ELISA and qPCR.

Test Result	Free-Range Cats	Total
ELISA (+)	262 (43.1%)	
ELISA (−)	346 (56.9%)	608
qPCR (+)	25 (4.2%)	
qPCR (−)	576 (95.8%)	601 ^1^
ELISA (+) and qPCR (+)	11 (1.8%)	
qPCR (+) and ELISA (−)	14 (2.3%)	
ELISA (+) and qPCR (−)	247 (41.1%)	
ELISA (−) and qPCR (−)	329 (54.7%)	601 ^1^

^1^ Whole blood was not available for DNA isolation in 7 cats (not tested by qPCR).

**Table 2:** Detection of *Cytauxzoon felis* by indirect ELISA and qPCR in the pet cat population.

	ELISA (+)	ELISA (−)	qPCR (+)	qPCR (−)	Total of Cats ^1^
**Lifestyle**					
Indoor	0	57	5	52	
Outdoor	1	15	2	14	
Both	3	24	5	22	
Subtotal	4	96	12	88	100
**Environment**					
Urban	0	6	1	5	
Suburban	1	18	0	19	
Rural	3	72	11	64	
Subtotal	4	96	12	88	100
**Tick prevention**					
Yes	3	33	4	32	
No	1	63	8	56	
Subtotal	4	96	12	88	100

^1^ Percentages are equal to N as the total of cats tested in this group is =100.

## Data Availability

The original contributions presented in this study are included in the article/Supplementary Material. Further inquiries can be directed to the corresponding author.

## Supplementary Materials

The following supporting information can be downloaded at https://www.mdpi.com/article/10.3390/vetsci13050426/s1, File S1: Client Questionnaire. Figure S1: ELISA absorbance values represented in percent positivity according to cat population; Figure S2: Percentage of ELISA positivity in experimentally infected cats, according to day PI; Table S1: Experimentally infected cats. *C. felis* detection day after tick infestation.

## Abbreviations

The following abbreviations are used in this manuscript:

ELISA	Enzyme-linked immunosorbent assay
qPCR	Quantitative polymerase chain reaction
ddPCR	Digital droplet polymerase chain reaction
PI	Post infection
k	1000
