# Supplementary material for: Utilization of qPCR and ELISA Tests to Detect Cytauxzoon felis (Theileriidae) in Domestic Cats (Felis catus) from South Central USA
Source: Vet Sci. 2026 Apr 28;13(5):426. doi: 10.3390/vetsci13050426 (PMC13211381; doi:10.3390/vetsci13050426)
Supplement: Supplementary file 1 [file vetsci-13-00426-s001.zip › Supplementary File S1.pdf]

Cat ID (Lab use only):

Client Questionnaire
Bobcat fever (*Cytauxzoon felis*) prevalence survey on pet cats

Collection date: __________**Client name**:________________________________(Not required) **Pet Name**: ____________

Do you consent to collect blood from your cat for research purposes? Yes   No

If yes, please select one from each of the following categories:

Gender: Male   Female   **Age:** < 6 months   6 - 12 months   1-3 years   > 3 years

Lifestyle: Indoor Only   Indoor/Outdoor   Outdoor Only

Environment: Rural (< 25,000)   Suburban (25,000-50,000)   Urban (>50,000)

Is the pet on flea/tick prevention?   Yes   No

- **If yes, please list which one:** ____

Email address to submit results: ____

Cat ID (Lab use only):

Client Questionnaire
Bobcat fever (*Cytauxzoon felis*) prevalence survey on pet cats

Collection date: __________**Client name**:________________________________(Not required) **Pet Name**: ____________
Do you consent to collect blood from your cat for research purposes? Yes   No
Please select one from each of the following categories:

Gender: Male   Female   **Age:** < 6 months   6 - 12 months   1-3 years   > 3 years

Lifestyle: Indoor Only   Indoor/Outdoor   Outdoor Only

Environment: Rural (< 25,000)   Suburban (25,000-50,000)   Urban (>50,000)

Is the pet on flea/tick prevention?   Yes   No

- **If yes, please list which one:** ____

Email address to submit results: ____
