# Supplementary figures and images for: Utilization of qPCR and ELISA Tests to Detect Cytauxzoon felis (Theileriidae) in Domestic Cats (Felis catus) from South Central USA
Source: Vet Sci. 2026 Apr 28;13(5):426. doi: 10.3390/vetsci13050426 (PMC13211381; doi:10.3390/vetsci13050426)

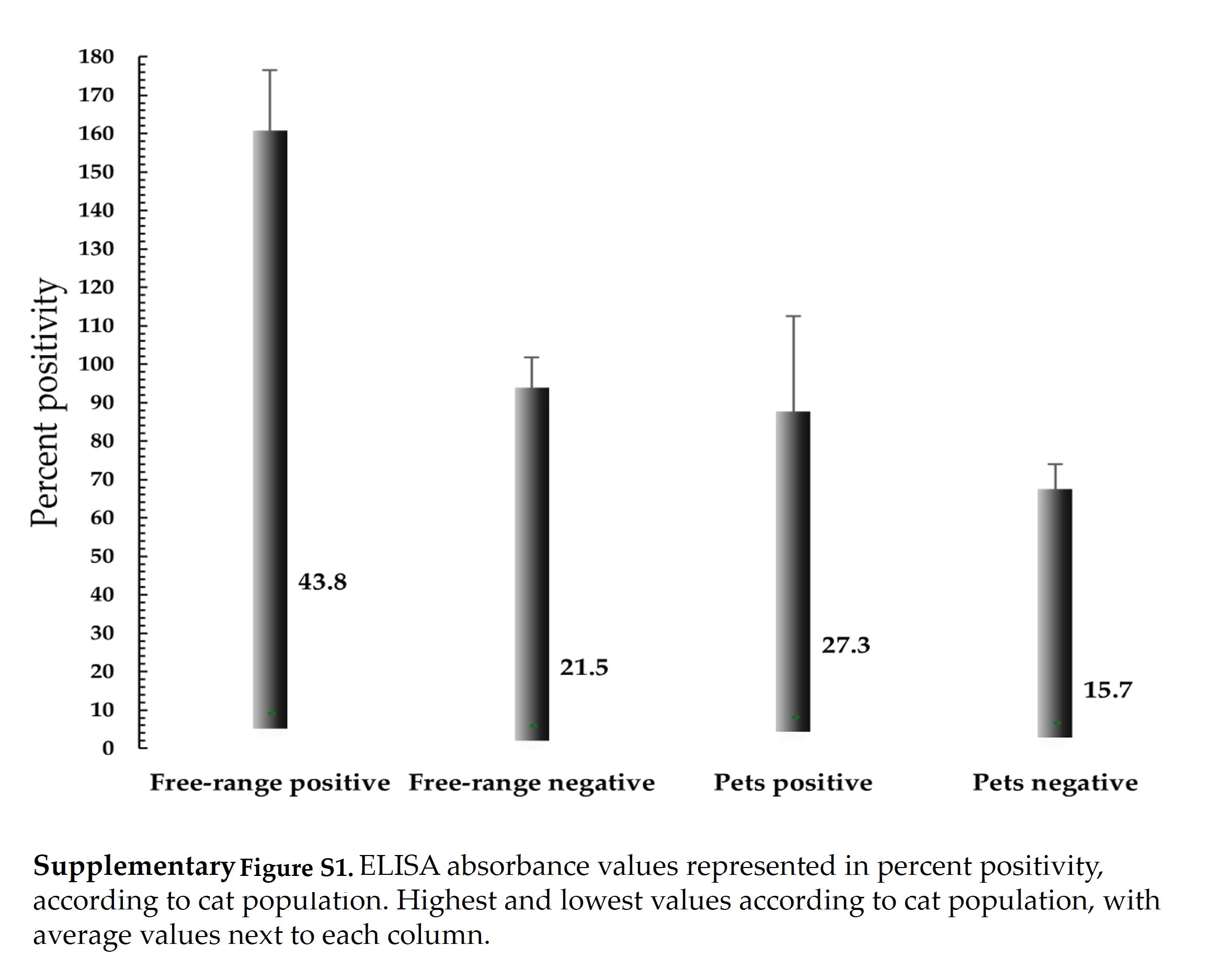

Supplement: Supplementary file 1 [file vetsci-13-00426-s001.zip › Supplementary Figure S1.jpg]

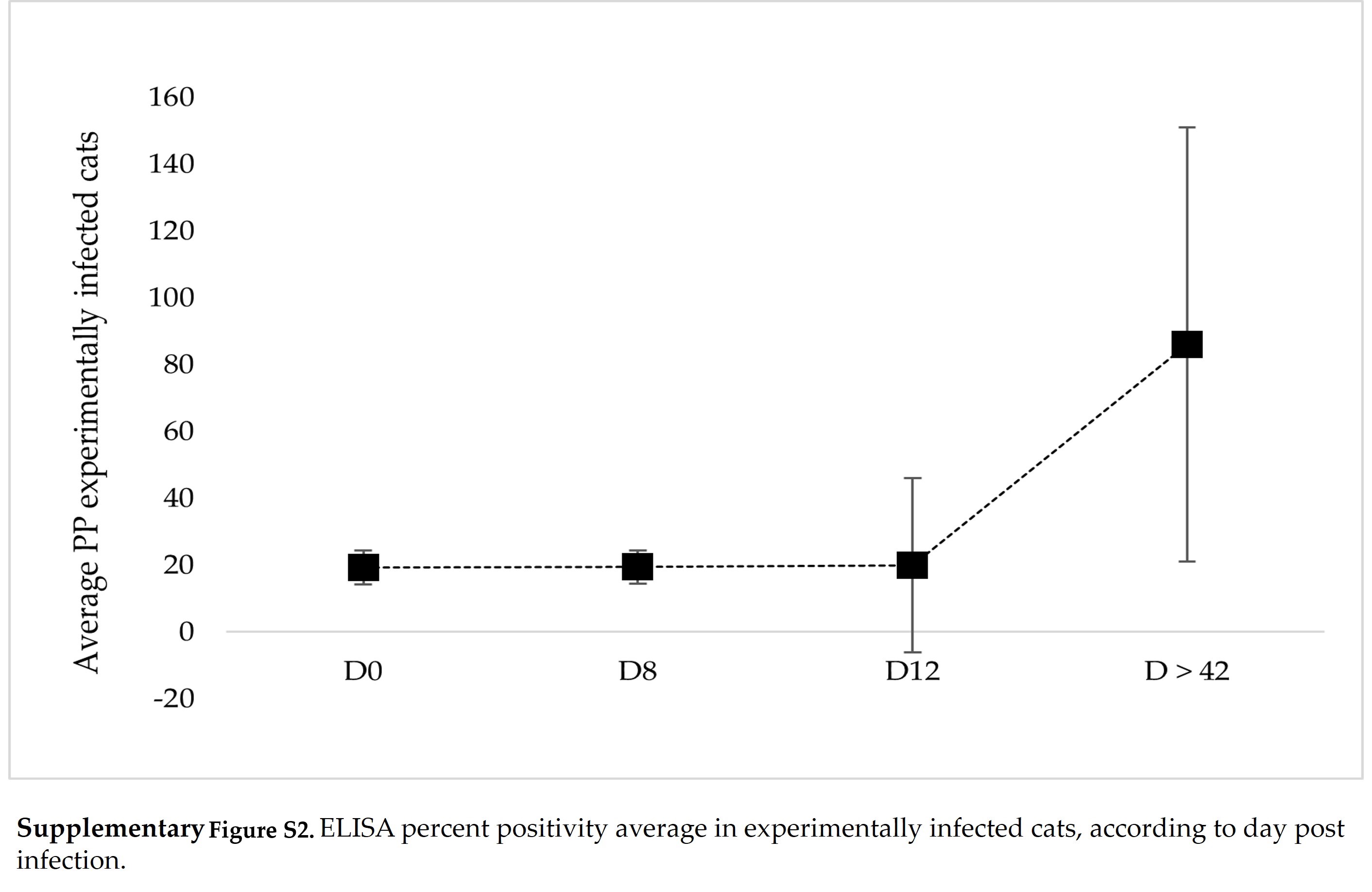

Supplement: Supplementary file 1 [file vetsci-13-00426-s001.zip › Supplementary Figure S2.jpg]
